# Effect of Water-Based vs. Land-Based Exercise Intervention (postCOVIDkids) on Exercise Capacity, Fatigue, and Quality of Life in Children with Post COVID-19 Condition: A Randomized Controlled Trial

**DOI:** 10.3390/jcm12196244

**Published:** 2023-09-28

**Authors:** Anna Ogonowska-Slodownik, Marta Kinga Labecka, Agnieszka Maciejewska-Skrendo, Renae J. McNamara, Katarzyna Kaczmarczyk, Michał Starczewski, Jan Gajewski, Natalia Morgulec-Adamowicz

**Affiliations:** 1 Faculty of Rehabilitation, Jozef Pilsudski University of Physical Education in Warsaw, Marymoncka 34, 00-968 Warsaw, Poland; marta.labecka@awf.edu.pl (M.K.L.); katarzyna.kaczmarczyk@awf.edu.pl (K.K.); michal.starczewski@awf.edu.pl (M.S.); jan.gajewski@awf.edu.pl (J.G.); natalia.morgulec@awf.edu.pl (N.M.-A.); 2 Faculty of Physical Culture, Gdansk University of Physical Education and Sport, 80-336 Gdansk, Poland; agnieszka.maciejewska-skrendo@awf.gda.pl; 3 Institute of Physical Culture Sciences, University of Szczecin, 70-453 Szczecin, Poland; 4 Physiotherapy, Prince of Wales Hospital, Sydney, NSW 2031, Australia; renae.mcnamara@health.nsw.gov.au; 5 Faculty of Medicine and Health, Sydney School of Health Sciences, The University of Sydney, Sydney, NSW 2006, Australia; 6 Woolcock Institute of Medical Research, Sydney, NSW 2037, Australia

**Keywords:** child, long COVID, SARS-CoV-2, exercise capacity

## Abstract

Evidence suggests that COVID-19 can cause lasting health consequences called post-COVID-19 condition. We conducted a three-group, randomized controlled trial for children aged 10–12 years with post COVID-19 condition. Participants were randomized to AQUA, LAND, or CONTROL groups. The AQUA and LAND training sessions were conducted twice a week for eight weeks. The primary outcomes were exercise capacity, measured using the modified Balke treadmill protocol, and fatigue, measured using the Cumulative Fatigue Symptoms Questionnaire (CFSQ). The secondary outcome was health-related quality of life (HRQoL), measured with the Pediatric Quality of Life Inventory (PedsQL) for children and parents. A total of 74 of the 86 children completed the intervention and attended the post-intervention assessment. The absolute maximal oxygen uptake (VO_2_max) values increased after both AQUA (*p* = 0.001) and LAND (*p* = 0.004) interventions. No significant differences were found in the degree of total fatigue and individual fatigue symptoms. A significant improvement was found in the PedsQL reported by the parents in the LAND group. In conclusion, the applied eight-week water-based and land-based exercise training programs improved exercise capacity in children aged 10–12 years old with post COVID-19 condition. The parents of the children in the training groups also noted an improvement in HRQoL.

## 1. Introduction

Evidence suggests that COVID-19 can cause lasting health consequences after the acute phase of the infection. Post COVID-19 condition occurs in individuals with a history of confirmed or probable SARS-CoV-2 infection when experiencing symptoms lasting at least 2 months which initially occurred within 3 months of acute COVID-19 [1]. The most common symptoms of post COVID-19 condition in children are fatigue, anxiety, post-exertional malaise, weakness, and dyspnea [2,3,4,5]. Although many children infected with SARS-CoV-2 experience mild symptoms or none at all, evidence suggests that up to 66% of children recovering from COVID-19 can face permanent and recurrent health problems [6,7,8,9,10]. Therefore, it is very important not to underestimate the long-term consequences on children and provide them with comprehensive diagnostics, care, and support.

Rehabilitation plays a key role in treating post COVID-19 condition, with a focus on improving respiratory and motor functions. Effective strategies use pulmonary rehabilitation to address the impaired lung ventilation function, muscle weakness, and reduced exercise tolerance [11] occurring after COVID-19; these have the aim of improving respiratory symptoms, reducing complications and disabilities, preserving function, and positively influencing the psychological sphere in adults [12,13]. However, variety in the severity and duration of post COVID-19 symptoms in children pose significant challenges to precise recommendations for exercise.

Water-based and land-based exercise interventions are safe and have a positive influence on physical conditions in children. Exercise performed in the aquatic environment has been found to improve exercise capacity, pulmonary function, and quality of life in children, by exploiting the hydrostatic pressure, temperature, buoyancy, turbulence, and resistance of the water [14]. Alternatively, land-based exercises in the form of active play exercises, team games, circuit training, and pair exercises are associated with satisfaction, high attendance rate, and relatively high exercise intensity in children with asthma [15].

As COVID-19 can have a long-term impact on a child’s health, there is a need to implement and evaluate therapeutic interventions that can effectively treat the symptoms of post COVID-19 condition in children. However, little if any formal research has been performed in this area to date. Therefore, the purpose of this study was to determine the effectiveness of water-based and land-based exercise training programs on exercise capacity, fatigue, and health-related quality of life in children with post COVID-19 condition.

## 2. Materials and Methods

### 2.1. Design and Setting

We conducted a three-group randomized controlled trial. Children with post COVID-19 condition were recruited from primary care clinics and schools in Warsaw (Poland) between January and March 2022. The trial was prospectively registered on ClinicalTrials.gov (NCT05216549, registration date 31 January 2022), and the protocol has previously been published elsewhere [16]. There were no changes in the methods and outcomes after the trial commencement. The study was conducted in accordance with the Declaration of Helsinki and approved by the Ethics Committee of Jozef Pilsudski University of Physical Education in Warsaw (protocol code 01-55/2021, date of approval 12 January 2022). Parents of the participants completed a written informed consent before initiating study procedures.

### 2.2. Participants

The inclusion criteria comprised age of 10–12 years and symptoms typical of post COVID-19 condition, including fatigue and shortness of breath/respiratory issues, at least one month after an initial COVID-19 infection. The exclusion criteria consisted of the following: absolute contraindications to exercise, unstable cardiac conditions, and currently engaged in regular exercise training more than twice per week. The parents of the potential participants answered an online screening questionnaire. If eligible, and having provided written consent, the children were assessed by the general practitioner. Pre-participation screening for exercise testing and training with the general practitioner included: a medical history questionnaire, general health screen, blood pressure, medication use, nutritional assessment, heat and hydration-related risk factors, mental health considerations, and test for SARS-CoV-2 antibody. The necessary minimum total number of participants (n = 69) was determined using G*Power software 3.1.9.7, assuming the detection of a moderate effect size of REPETITION×GROUP interaction (d = 0.50 or eta square 0.04) for exercise capacity and fatigue with a significance level of 0.05 and statistical power of 0.85.

### 2.3. Randomization, Allocation Concealment and Blinding

Participants meeting the inclusion criteria were randomized into one of three groups: water-based exercise (AQUA), land-based exercise (LAND), or CONTROL (no exercise). The randomization sequence was designed by an investigator external to the study using an Excel random number generator. Group allocation was prepared in 5 random versions. The allocation providing the lowest F value for MANOVA comparing the basic characteristics of the study subjects was used. Randomization was performed after the baseline assessment session and stratified according to age, sex, and the level of exercise capacity. Concealed allocation was achieved using opaque envelopes. Due to the nature of exercise interventions, it was not possible to blind the physiotherapist or participants.

### 2.4. Intervention

The AQUA and LAND group training sessions were conducted twice a week, 45 min per session, for eight weeks. The programs were delivered in locations in the community that were easily accessible. The exercises were supervised by experienced physiotherapists. Both the AQUA and LAND interventions have been described in detail in the published trial protocol [16]. In brief, the AQUA sessions were conducted in a swimming pool (depth 1.2–1.5 m; length 12 m; width 3 m; water temperature 30 °C; humidity 60%). The participants were encouraged to exercise at a depth where the water level sat between the clavicle and xiphisternum. The LAND sessions were conducted in the gym with a controlled temperature.

Both the water and land exercises were matched as closely as possible in terms of intensity, duration, and muscle groups trained. Each session consisted of a warm-up (8 min), aerobic training (32 min), and a cool down (5 min). Warm-up consisted of upper- and lower-limb aerobic exercises including punching and kicking; jogging (stationary); breathing control; and lower-limb stretches. The aerobic training component consisted of two circuit stations with endurance exercises for the upper and lower limbs and a focus on breathing patterns. Each circuit comprised of five stations with different exercises. The activity at each station lasted one minute, with 15-s rest periods in between. Participants were encouraged to exercise at an intensity of 6–8 (“getting quite hard” to “hard”) on the Pictorial Children’s Effort Rating Table (PCERT) [17]. Cool down consisted of upper-limb and thoracic cage stretches and breathing control. The CONTROL (no exercise) group was advised to not alter their current exercise routine, and was offered the option of joining an AQUA or LAND exercise program after completion of the final assessment.

### 2.5. Primary Outcomes

#### 2.5.1. Exercise Capacity

Exercise capacity was measured using the modified Balke treadmill protocol [18]. The first three minutes consisted of walking on the flat without an incline, with the speed set at 5.3 km/h, to become familiar with the treadmill. After the first three minutes, the incline of the treadmill increased to 6%, and then by 2% each minute until 22%. After reaching 22%, the angle of the treadmill remained constant and each minute the treadmill speed increased by 0.5 km/h until volitional exhaustion. Standardized verbal encouragement was given throughout the test. Heart rate was monitored continuously using the POLARV800 (Polar Electro OY, Helsinki, Finland). Breathing parameters were measured by the “breath-to-breath” method using the Cortex MetaMax 3B ergospirometer system (Biophysik GmbH, Leipzig, Germany). The gas analyses were sampled at 15-s intervals. The maximal oxygen uptake values (VO_2_max) were established from the highest achieved intensity 15-s samples. The oxygen uptake efficiency slope (OUES) was calculated from the collected data [18].

#### 2.5.2. Fatigue

Fatigue was measured using the validated Polish adaptation of the Cumulative Fatigue Symptoms Questionnaire (CFSQ) for adolescents, also named the Cumulative Fatigue Symptoms Index [19]. This questionnaire measures the level of chronic fatigue and the severity of individual symptoms including general fatigue, decreased vitality, mental overload, somatic symptoms, anxiety, and discouragement about studying and school. The complaint rate (CR) for each symptom was calculated as follows: CR (%) = (number of positive items)/(number of items × number of participants) × 100.

### 2.6. Secondary Outcome

Health-related quality of life (HRQoL) was measured with the validated Polish version of the Pediatric Quality of Life Inventory 4.0 Generic Core Scales (PedsQL) for children and parents [20]. Both participants and their parents completed the questionnaire separately.

### 2.7. Procedure

At the baseline assessment session, participants and parents were requested to complete the PedsQL 4.0 and CFSQ. The research team measured the participant’s weight and height and calculated body mass index (BMI). The general practitioner examined the participants to determine any contraindications to exercise testing and after that exercise capacity measurement was performed. The same evaluation was performed after the end of the eight-week training program.

### 2.8. Statistical Methods

Statistical analyses were completed using Statistica 14 (TIBCO Software Inc., Palo Alto, CA, USA 2020, Data Science Workbench, version 14, http://tibco.com, accessed on 1 February 2023). For interactions between repeated and fixed factors, it was assumed that medium-sized effects (partial eta square = 0.06) were to be detected at a significance level of 0.05 and a statistical power of 0.85. The normality of the distributions within groups of continuous variables was tested using the Shapiro–Wilk test. As the distributions were normal, the means were determined by analysis of variance for repeated measures. Group (CONTROL, AQUA, LAND) and SEX (MALE, FEMALE) were included as fixed factors while MEASUREMENT (PRE, POST) was analyzed as a repeated factor. Homogeneity of variance was assessed using Levene’s test, and Tukey’s HSD test was used for detailed post-hoc comparisons. Quantitative data were presented as mean ± standard deviation (SD) together with a 95% confidence interval (CI).

Ordinal variables were compared using non-parametric methods. The groups were compared using the Kruskal–Wallis test, and the pre- and post-intervention values using the Wilcoxon matched pairs test. Between-group comparisons were made using the Mann–Whitney test. Associations between variables were assessed using Spearman rank correlation. The level of agreement between the responses from children and parents in the PedsQL questionnaire was analyzed using Cohen’s kappa; the kappa values were interpreted based on data according to Landis & Koch [21]. The level of significance was set at α = 0.05. Effect sizes were estimated using partial eta squared (ANOVA), and equivalent correlation coefficient (Wilcoxon test). Missing data were pairwise deleted.

## 3. Results

**Figure 1:**
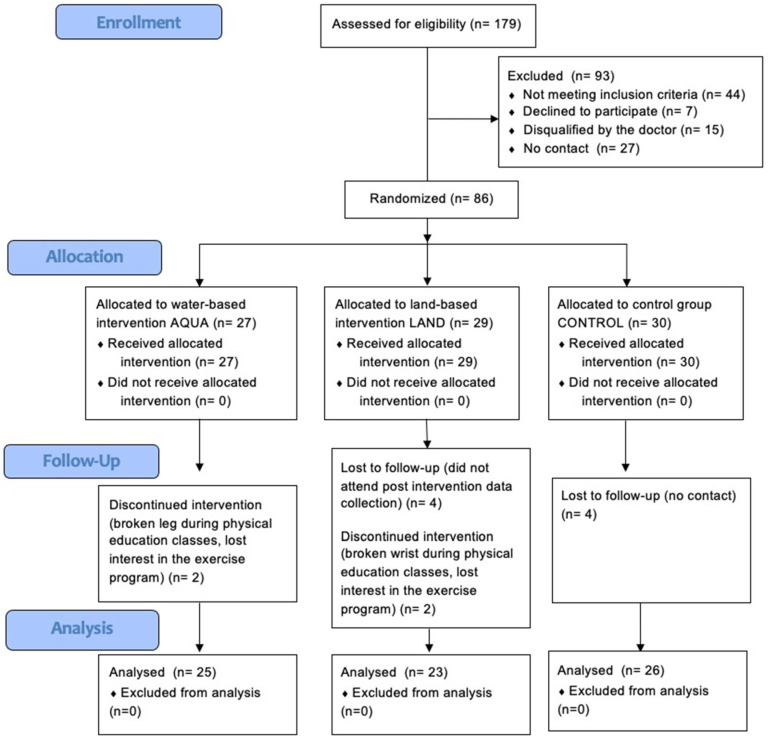
CONSORT flow diagram of participants.

Recruitment began in January 2022, and the post-intervention data were collected in June 2022. The flow of participants through the trial is shown in Figure 1. Of the 179 children assessed for eligibility, 86 met the inclusion criteria and were randomized. Seventy-four children (86%) completed the intervention and attended the post-intervention assessment. The mean attendance rate was 84% in the AQUA group and 77% in the LAND group. There was no significant difference in attendance between the two groups, and no adverse events were recorded.

No significant differences were found between groups before the intervention for any measured parameter (Table 1).

### 3.1. Primary Outcomes

An increase in relative VO_2_max values was observed in only the AQUA group after eight weeks of intervention (*p* = 0.017) (Table 2).

**Figure 2:**
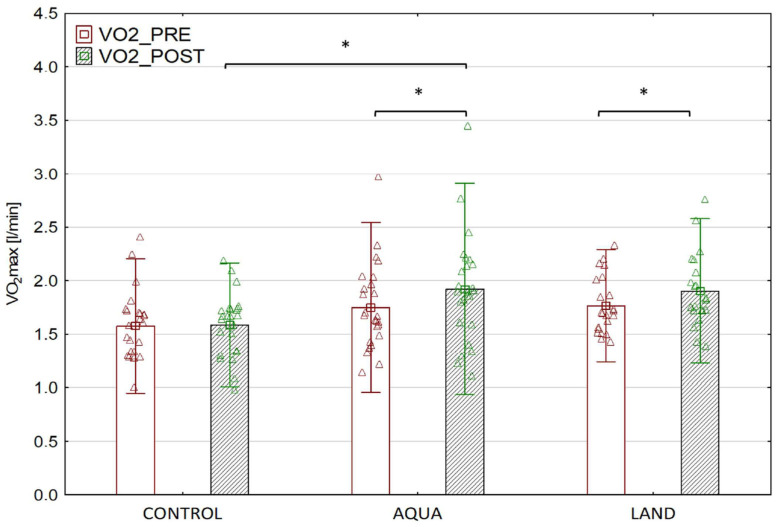
Maximal oxygen uptake in each group before and after the intervention. * *p* < 0.05.

The within-group comparisons found the absolute values of VO_2_max to increase after both types of intervention, AQUA (*p* = 0.001) and LAND (*p* = 0.004), but not in the CONTROL group (*p* = 0.999). The only significant difference in post-intervention VO_2_max values was observed between the AQUA and CONTROL groups (*p* = 0.023) (Figure 2).

No significant differences were found among the three groups regarding the change in total fatigue score or individual fatigue symptoms (complaint rate) from before and after the intervention, according to the CFSQ (Supplementary Table S1). All three groups demonstrated different distributions of fatigue levels (low: scores 0–14 pts, moderate scores 15–30 pts, and high scores 31–45 pts) pre and post-intervention (Table 3). No “very high” fatigue scores, i.e., from 46 to 60 pts, were reported.

### 3.2. Secondary Outcome

The overall HRQol, indicated by the total PedsQL score, significantly improved only in the LAND group of parents (*p* < 0.01). There was no between-group effect in the PedsQL total score neither in children nor in parents (Table 2). Significant improvements in physical, emotional, social, and school functioning were reported only by the parents in the LAND group (respectively, *p* < 0.01; *p* < 0.01; *p* < 0.05; *p* < 0.05). In the AQUA and LAND groups, the parents reported minimally clinically meaningful differences in the total PedsQL score (4.85 and 9.83 respectively), as estimated by Varni et al. [22]. (Supplementary Table S2).

No agreement was found between the PedsQL scores returned by the children and parents before the intervention, in either the AQUA, LAND, or CONTROL groups; however, after the intervention, slight agreements were evident between children and parents for physical functioning (κ = 0.097; *p* < 0.001), social functioning (κ = 0.145; *p* < 0.001) and school functioning (κ = 0.101; *p* < 0.003).

Significant correlations were found between overall HRQoL and total fatigue score, before and after intervention, for the AQUA group (respectively, r = −0.85, *p* < 0.001; r = −0.89, *p* < 0.001), LAND group (respectively, r = −0.68, *p* < 0.001; r = −0.76, *p* < 0.001) and CONTROL group (respectively, r = −0.74, *p* < 0.001; r = −0.89, *p* < 0.001).

## 4. Discussion

This study is the first randomized controlled trial to examine the effect of supervised exercise training programs in children with post COVID-19 condition. The results indicate that exercise capacity is improved after both AQUA and LAND exercise training, with the most significant improvement in VO_2_max observed following water-based exercise training.

Numerous studies have demonstrated that aerobic exercise improves the overall physical performance of patients with respiratory diseases, such as chronic obstructive pulmonary disease or pulmonary fibrosis [23,24]. Like those suffering from post COVID-19 condition, these patients also demonstrate dyspnea and shortness of breath [25,26,27]. Progressive dyspnea and prolonged periods of shortness of breath can lead to fatigue and decreased cardiopulmonary fitness, exercise capacity, and physical activity [28]. Many studies have shown that improving skeletal muscle function through physical activity in the form of exercise programs performed on land or in water has a positive effect on alleviating symptoms associated with impaired respiratory function [29,30,31,32]. The most effective programs for improving quality of life and exercise capacity are those using strength training or cardiovascular interval training [33,34]. Our findings do not show that the training interventions had a direct positive effect on post COVID-19 symptoms, as no significant improvement was noted in fatigue indicators. However, a significant improvement in VO_2_max values was found, indicating that training had a positive effect on improving the overall respiratory capacity of the children.

Land-based exercise training programs are typically easy to implement in locations with suitable open spaces or in facilities with access to exercise machines; as such, they are relatively accessible and inexpensive. However, land-based exercise training is not always suitable for people with medical and physical co-morbid conditions such as permanent skeletal muscle dysfunction, chronic joint pain, or balance disorders, and often the observed rehabilitation effects have been less than expected [35,36,37]. In the aquatic environment, the specific characteristics of the water seem to have a particularly beneficial effect and can improve the effectiveness of exercise in people with physical co-morbid conditions such as musculoskeletal disease [31]. The unique properties of the water contribute to the relief of a variety of symptoms by strengthening muscles [38]; these have been shown to ameliorate the symptoms often experienced with co-morbid conditions [39]. Moreover, exercise in water increases venous return and positively affects cardiorespiratory fitness [40]. In addition, the element of novelty and attractiveness of water exercises [41], as well as their differences with more frequently performed land-based activities, is an important element for engaging younger people in exercise training. Indeed, in the present study, the AQUA group was characterized by greater attendance. We hypothesized that the more pleasant aquatic environment was more acceptable to children, and the novelty factor led to greater engagement. Our data also indicates that the AQUA training improved exercise capacity to a greater degree than the LAND training; it is possible that the characteristics of the aquatic environment forced greater ventilation and higher energy expenditure compared to the land.

Interestingly, the analysis of self-reported fatigue did not reveal a high level of this symptom in the children in the study. Although a positive tendency was observed, with more children reporting a lower level of fatigue after the intervention. Even though fatigue is one of the most common symptoms reported in post COVID-19 condition, most children included in the study presented with a low or moderate level of fatigue at initial assessment, hence it was difficult to see a further improvement in fatigue from already low baseline levels. One of the reasons for this could be the partial inadequacy of the tool used to measure fatigue. Although the CFSQ has been used in adolescents, it may have been too complicated for 10–12-year-old children, which may have contributed to an underestimation of their fatigue.

In young children, the best practice to assess HRQoL demands the combined use of questionnaires or interview data from multiple informants, such as parents, teachers, clinicians, and child self-reports [42]. In the present study, the children completed the HRQoL questionnaire separately from their parent. Although no agreement between the child and parent questionnaire scores before the intervention, a slight agreement was noted between the child and parent after the intervention. Levels of agreement can be affected by the child’s age, the domains investigated, and the parents’ quality of life [43]. Furthermore, a significant post-intervention improvement in HRQoL was found in the questionnaire results, but only by parents in the LAND group. The probable cause of such a result could be the specific design of the PedsQL instrument; it is possible that the question-answer scheme used with content specific to land-based activities, such as walking or running, made it more difficult for participants exercising in the water to notice improvement in skills specific to land. A minimal clinically significant difference was observed in the total score of the PedsQL reported by the parents in both the AQUA and LAND groups.

This study has demonstrated that both land-based and water-based supervised exercise training programs are effective and feasible for children aged 10–12 years old. The 45-minute duration of the session, frequency twice a week, and eight-week length of the exercise programs were well accepted, there was high attendance, and the programs were safe with no adverse events.

### Limitations

The lack of any specific diagnostic criteria for post COVID-19 condition resulted in a heterogeneous group of participants. The lack of specific measurement tools designed and validated in children with post COVID-19 condition was a limitation in adequately assessing fatigue and HRQoL. Another limitation of the study is the lack of cognitive assessment. In addition, the relatively small study sample size limits the interpretation of the secondary outcome of HRQoL results. Finally, the long-term effects of exercise training were not included in this study, and future studies should examine those effects in children with post COVID-19 condition.

## 5. Conclusions

In children aged 10–12 years old with post COVID-19 condition, an eight-week, twice-weekly supervised water-based or land-based exercise training program improved exercise capacity. No effect on fatigue was found, and no adverse events were observed. HRQoL improvement was observed by the parents of the children in the training groups.

## Figures and Tables

**Table 1:** Pre-intervention characteristics for the AQUA, LAND, and CONTROL groups.

	AQUA (n = 25)	LAND (n = 23)	CONTROL (n = 26)
Female, n (%)	15 (60)	14 (61)	14 (54)
Age (years)	10.8 (0.8)	11.0 (0.7)	10.6 (1.0)
Body mass (kg)	45.2 (14.1)	43.4 (1.6)	38.3 (9.5)
Height (m)	1.52 (0.1)	1.52 (0.1)	1.46 (0.1)
BMI (kg/m^2^)	19.3 (4.6)	18.7 (3.7)	17.9 (3.6)

Data presented as mean (SD) unless otherwise stated. BMI, body mass index.

**Table 2:** Between-group comparisons for exercise capacity, fatigue, and health-related quality of life.

		AQUA	LAND	CONTROL	F_2,64_	*p*	ƞ^2^	Between-Group Difference Post-Intervention (95% CI)		
								AQUA vs. LAND	AQUA vs. CONTROL	LAND vs. CONTROL
		(n = 25)	(n = 23)	(n = 26)						
**VO_2_max (mL/kg/min)**	Pre	40.0 (7.0)	42.9 (8.3)	41.6 (6.9)	4.32	0.017	0.11	2.9 (−1.5 to 7.3)	1.6 (−2.3 to 5.5.)	1.3 (−3.1 to 5.7)
	Post	42.6 (7.5) ^a^	45.5 (7.8)	41.2 (6.8)				2.9 (−1.5 to 7.4)	1.4 (−2.6 to 5.4)	4.3 (0.01. to 8.6)
**VE (L/min)**	Pre	61.4 (17.4)	60.7 (9.8)	54.3 (11.0)	9.60	0.001	0.22	0.7 (−7.6 to 8.9)	7.1 (−1.1 to 15.3)	6.4 (0.4 to 12.5)
	Post	69.6 (18.3) ^a^	68.7 (13.0) ^b^	53.1 (10.7) ^c,d^				0.9 (−8.5 to 10.2)	16.5 (8.0 to 24.9)	15.6 (8.7 to 22.5)
**HRmax (beats/min)**	Pre	189 (14)	195 (6)	190 (14)	2.90	0.062	0.08	6.1 (−0.2 to 12.5)	1.0 (−6.7 to 8.8)	5.1 (−1.0 to 11.3)
	Post	192 (12)	191 (17)	187 (13)				1.8 (−6.9 to 10.6)	5.4 (−1.6 to 12.6)	3.6 (−5.5 to 12.8)
**OUES (L/min)**	Pre	2.14 (0.5)	2.29 (0.5)	2.08 (0.5)	2.95	0.059	0.08	0.1 (−0.1 to 0.5)	0.1 (−0.2 to −0.3)	0.2 (−0.1 to 0.5)
	Post	2.27 (0.6)	2.23 (0.5)	2.05 (0.5)				0.04 (−0.3 to 0.4)	0.2 (−0.1 to 0.5)	0.2 (−0.1 to 0.5)
**OUES (mL/kg/min)**	Pre	48.6 (7.9)	55.0 (11.3)	55.0 (11.6)	1.97	0.147	0.06	6.3 (0.8 to 11.9)	6.4 (0.8 to 11.9)	0.03 (−6.6 to 6.7)
	Post	50.1 (7.8)	52.8 (9.6)	52.8 (10.6)				2.7 (−2.3 to 7.8)	2.7 (−2.5 to 7.9)	0.04 (−5.9 to 6.0)
**RER**	Pre	0.98 (0.1)	0.99 (0.1)	0.98 (0.1)	0.48	0.623	0.01	0.006 (−0.03 to 0.04)	0.004 (−0.03 to 0.04)	0.01 (−0.01 to 0.03)
	Post	1.02 (0.05)	1.02 (0.03)	1.00 (0.04)				0.003 (−0.02 to 0.03)	0.02 (−0.003 to 0.05)	0.02 (−0.003 to 0.04)
**Total fatigue (pts)**	Pre	16.9 (9.8)	15.3 (8.0)	15.1 (8.1)	0.165	0.848	0.005	1.6 (−3.5 to 6.7)	1.8 (−3.1 to 6.7)	0.2 (−4.3 to 4.7)
	Post	15.1 (8.5)	13.8 (8.5)	14.2 (9.4)				1.2 (−3.6 to 6.1)	0.8 (−4.1 to 5.8)	0.4 (−4.6 to 5.4)
**Total score PedsQL children**	Pre	74.6 (13.1)	77.3 (11.6)	76.1 (9.7)	0.324	0.724	0.009	2.6 (−4.4 to 9.6)	1.5 (−4.8 to 7.8)	1.1 (−4.8 to 7.1)
	Post	74.3 (13.3)	78.7 (11.9)	77.1 (12.2)				4.3 (−2.8 to 11.5)	2.7 (−4.3 to 9.7)	1.6 (−5.2 to 8.4)
**Total score PedsQL parent**	Pre	66.5 (13.6)	68.7 (13.5)	70.6 (17.7)	2.449	0.094	0.067	2.2 (−5.6 to 9.9)	4.0 (−4.7 to 12.8)	1.9 (−7.2 to 10.9)
	Post	71.9 (11.9)	79.2 (9.9) ^b^	71.8 (17.2)				7.2 (0.9 to 13.5)	0.2 (−8.1 to 8.4)	7.4 (−0.6 to 15.4)

Data presented as mean (SD) unless otherwise stated. ^a^—significantly different from AQUA pre-intervention. ^b^—significantly different from LAND pre-intervention. ^c^—significantly different from AQUA post-intervention. ^d^—significantly different from LAND post-intervention. VO_2_max, maximal oxygen uptake; VE, minute ventilation; HRmax, maximal heart rate; OUES, oxygen uptake efficiency slope; RER, maximal respiratory equivalent ratio.

**Table 3:** Distribution of the total fatigue scores across fatigue levels according to the CFSQ for AQUA, LAND, and CONTROL groups (n/%).

	Fatigue Level					
	Low (0–14 Points)		Moderate (15–30 Points)		High (31–45 Points)	
	PRE (n/%)	POST (n/%)	PRE (n/%)	POST (n/%)	PRE (n/%)	POST (n/%)
LAND (n = 23)	8/35	12/52	14/61	10/44	1/4	1/4
AQUA (n = 25)	11/44	14/56	11/44	10/40	3/12	1/4
CONTROL (n = 26)	15/58	17/65	9/34	6/23	2/8	3/12

No significant correlations were found between total fatigue scores and VO_2_max, pre and post-intervention, in the groups.

## Data Availability

Available upon request. Proposals should be directed to anna.ogonowskaslodownik@awf.edu.pl.

## Supplementary Materials

The following supporting information can be downloaded at: https://www.mdpi.com/article/10.3390/jcm12196244/s1, Table S1: Cumulative Fatigue Symptoms Questionnaire (CFSQ) for adolescents: complaint rate (%) of individual symptoms severity including general fatigue, decreased vitality, mental overload, somatic symptoms, anxiety and discouragement; Table S2: Pediatric Quality of Life Inventory 4.0 Generic Core Scales (PedsQL) for parents: physical, emotional, social, school functioning and the total score of the PedsQL.
