# Supplementary material for: Effect of Water-Based vs. Land-Based Exercise Intervention (postCOVIDkids) on Exercise Capacity, Fatigue, and Quality of Life in Children with Post COVID-19 Condition: A Randomized Controlled Trial
Source: J Clin Med. 2023 Sep 28;12(19):6244. doi: 10.3390/jcm12196244 (PMC10573606; doi:10.3390/jcm12196244)
Supplement: Supplementary file 1 [file jcm-12-06244-s001.zip › jcm-2620672-supplementary.pdf]

Journal of
Clinical Medicine

Table S1. Cumulative Fatigue Symptoms Questionnaire (CFSQ) for adolescents: complaint rate (%) of individual symptoms severity including general fatigue, decreased vitality, mental overload, somatic symptoms, anxiety and discouragement.

	Group					
	AQUA (n=25)		LAND (n=23)		CONTROL (n=26)	
	PRE	POST	PRE	POST	PRE	POST
**General fatigue**	61	60	60	57	54	49
**Decreased vitality**	38	40	37	35	42	35
**Mental overload**	57	50	50	28	55	54
**Somatic symptoms**	34	33	30	33	13	18
**Anxiety**	50	44	45	30	36	38
**Discouragement about studying and school**	39	34	37	32	45	42

Data presented as complaint rate (%) of individual symptoms experienced by participants in AQUA, LAND and CONTROL groups.

Table S2. Pediatric Quality of Life Inventory 4.0 Generic Core Scales (PedsQL) for parents: physical, emotional, social, school functioning and the total score of the PedsQL.

	Group					
	AQUA (n=25)		LAND (n=23)		CONTROL (n=26)	
	PRE	POST	PRE	POST	PRE	POST
**Physical Functioning**	67.5 (17.8)	72.9 (17)	68.9 (15.3)	81.7 (13)**	70.6 (18.8)	74.2 (18.2)
**Emotional Functioning**	57.2 (19.5)	61.5 (16.5)	59.5 (18.9)	70.2 (14.3)**	58.2 (20.3)	64.2 (21.5)
**Social Functioning**	77.2 (17.3)	81.7 (13.7)	80.2 (16.4)	88.3 (12.5)*	82 (22.1)	79.4 (20.5)
**School Functioning**	63.8 (17.1)	71.3 (16.4)	66.1 (17.7)	75 (15.4)*	71.6 (21.3)	67.9 (22.4)
**Total score PedsQL™**	66.6 (13.6)	72 (11.9) †	68.7 (13.5)	79.2 (9.9)** †	70.6 (17.7)	71.8 (17.2)

Data presented as complaint mean (SD); ** $p < 0.01$; * $p < 0.05$; † minimal clinically meaningful difference (PRE-POST change > 4.5).
